# 

**Published:** 1915-07

**Authors:** 


### INDEX FOR VOLUME 70

### AUGUST, 1914, to JULY, 1915

| Page | | Page | |
|---|---|---|---|
| 1— 68 | August | 391—448 | February |
| 69—136 | September | 449—502 | March |
| 137—200 | October | 502—556 | April |
| 201—266 | November | 551—610 | May |
| 267—332 | December | 611—662 | June |
| 333—390 | January | 663—716 | July |

### CONTRIBUTORS:

BAUM, H. C., Syracuse, The Urine in Skin Diseases, Dec., page 267.

BRITT, F. Warren, Tonawanda, Pabulum, Nov., page 214.

BRUNDAGE, F. E., Buffalo, Aim of the Milk Station, Dec., page 281.

BURKE, Joseph, Buffalo, Diagnosis of Cancer of the Colon, Oct., page 137.

CANDEE, Pierce J., Buffalo, Anaemia, An Undescribed Cause, Its Easy Detection, April, page 507

CLEMESHA, John C., Buffalo, Congenital Word Blindness with Inability to Learn to Read, Aug., page 1.

COLTON, Albert J., Buffalo, Conditional versus Unconditional Quarantining for Acute Infectious Diseases which Confer to Persons Having them, Immunity for Life, Feb., page 399.

COTT, Chester C., Buffalo, Inflammatory Tonsillar Disease and its Cure, Oct., page 148.

COTT, George F., Buffalo, Some Affections of the Oesophagus, August, page 10.

COTTIS, G. W., Jamestown, Present Status of the Goitre Problem, Mar., page 449.

CROTHERS, T. D., New Haven, A New Field of Practice, November, page 209.

FRONCZAK, Francis E., Buffalo, First Free Municipal Bath Houses in America, June, page 658.

GAERTNER, Frederick, Pittsburg, Experimental, Pathologic and Vivisectional Proof that Cancer Incipiency is a Localized Chemo-hyperstimulated Toxic Lymph Process, Sept., page 69.

GARRATT, John M., Buffalo, X-Ray Therapy, Feb., page 391.

HANLEY, Lawrence G., Buffalo, Monodactylism, Oct., page 152.

HELD, Wm., Chicago, New Serum Treatment for Epilepsy, June, page 618.

HENNINGTON, Chas. W., Rochester, The Thymus Question, June, page 611.

HEPBURN, D. S., Buffalo, Chronic Lymphatic Leukaemia Complicated with Priapism, April, page 517.

HIGGINS, R. C., Cortland, Means of Early Treatment of Thyroid Hyperplasia, Dec., page 271.

LE BRETON, Prescott, Buffalo, Report of Unusual Cases of Ununited Fracture of Tibia, Repaired by Bone Grafting, Jan., page 344.

LEE, John M., Rochester, Clinical Report of Cancer Cases Treated with Radium, July, page 670.

LEO-WOLF, Carl G., Buffalo, Feeding of Infants and the General Practitioner, Nov., page 201.

LEWIS, James H., Buffalo, The Cancer Campaign, July, page 663.

LITTLE, Seelye W., Rochester, Cancer, An Approach from the Nutritional Side, July, page 679.

LOOP, Ross G., Elmira, Acute Pancreatitis with Report of a Case Successfully Diagnosticated and Operated, Sept., page 83.

MORRIS, Robert T., New York, Abdominal Cyst, Goitre, Hip Amputation, April, page 502.

MULLIGAN, E. W., and HENNINGTON, Chas. W., Rochester, Report of a Case of Ruptured Spleen, Sept., page 80.

NOEHREN, Alfred H., Buffalo, Transfusion of Blood, Jan., page 347.

PCHELLAS, V. A., Buffalo, Mixed Infection Phylacogen in the Treatment of Diabetic Gangrene, Aug., page 7.

QUINTON, W. W., Buffalo, Injuries of the Present War, May, page 557.

ROCHESTER, De Lancey, Buffalo, Angina Pectoris, Jan., page 333.

SPANGENTHAL, Jos., Buffalo, Electrolysis in Dermatology, Dec., page 278.

SPANGENTHAL, Jos., Buffalo, Pellagra, April, page 512.

VON OEFELE, Felix, New York, Natural Nitrated Mineral Waters of New England, Sept., page 77.

VON OEFELE, Felix, New York, Natural Silicated Mineral Springs of New England, Dec., page 286.

VON OEFELE, Felix, New York, Thermal Waters of the Eastern Half of the United States, Feb., page 405.

WARBRICK, John C., Chicago, Mucous Polypi in the Nose, Nov., page 221.

WARBRICK, John C., Chicago, Perforation of the Nasal Septum, May, page 563.

WOODRUFF, John V., Buffalo, President's Address, Medical Society of the County of Erie, N. Y., March, page 456.

**OBITUARY:**

Ainsworth, Herman R., Nov..... 245
Albertson, Charles S., Feb...... 431
Allen, William L., Mar.......... 498
Annowski, William H., Sept.... 120

Bacon, Charles J., Dec.......... 313
Bangs, Lemuel B., Nov.......... 240
Barnes, J. Franklin, Dec........ 312
Barney, Charles S., Aug........ 54
Beals, Fred. C. Oct.............. 195
Benton, Stuart H., April........ 555
Bettis, J. T., Jan................ 382
Braman, Charles B., Oct........ 195
Brown, John M., Aug............ 54
Brown, John W., Oct........... 195
Browne, Albert J., Mar.......... 498
Brownell, William G., May...... 609
Burgess, Maynard G., Nov...... 244
Busch, Frederick C., Dec........ 314
Button, Clayton A., June........ 657

Caldwell, H. W., April.......... 555
Calli, Angelo, Feb.............. 430
Campbell, George A., April...... 555
Carson, Matthew R., Nov....... 244
Campbell, C. Ernest, Jan........ 382
Cassidy, John J., Dec............ 313
Carpenter, L. W., Jan...........383
Clark, Edward D., Mar.......... 498
Clausen, Bernard, Nov.......... 246
Cobb, John W., Mar............ 498
Cook, Albert M., Nov............ 245
Cook, Joseph T., Feb............ 430
Cole, Directus D., July.......... 706
Corwin, Elizabeth, Dec.......... 313
Craton, Samuel B., April........ 555

Dann, Archibald M., Oct......... 196
Darrow, S. W., June............ 657
Davie, Frank C., Oct............ 194
Dee, John W., Sept............ 120
Devine, John R., Aug............ 54
Donoghue, James K., April...... 555

Earle, Exmon W., Oct.......... 194
Eckerson, Edward, Feb.......... 431
Elliott, Justin C., June.......... 657

Farrington, Dascum A., Sept.... 120
Foster, Daniel H., June......... 658
Foster, Gard, Jan................ 383
Fuller, C. M., June.............. 657
Fournier, Jean A., Feb.......... 430

Gail, William H., Mar.......... 497
Gates, Henry A., Aug........... 54
Gaviller, Edwin A., Dec........ 313
Gilbert, Horatio, June........... 657
Gouninlock, William C., Nov.... 244
Grant, John H., Sept............ 120

Graves, Newton, Feb............ 430
Gregory, George W., May...... 608
Guyott, Ezra J., Oct............. 195

Hart, Ira F., Jan................. 383
Hemstreet, James, Sept......... 122
Hill, Frank E., Mar............ 497
Hinman, Arthur F., Mar........ 498
Hubbard, Dwight G., Sept...... 120
Hubbell, Philo G., June........ 657
Hunter, William L., June........ 657
Hurlbut, Edwin T. M., June..... 657

Jackson, Andrew J., Jan........ 383
Jones Frank, Sept................ 121
Justin, Helen D., Sept.......... 120

Keator, Thomas O., Oct........ 195
King, James K., Nov............ 245
Koehler, Edward E., Feb........ 430
Kyle, Edmund H., Aug.......... 54

Lewis, Leon G., Dec............ 313
Loeffler, Friedrich, May......... 608

Magruder, Ernest P., May....... 608
Menzie, Robert J., June.......... 657
Mayer, Chester A., May........ 608
McClellan, Frederick E., May.... 608

Neff, Daniel D., Dec............ 313
Nichols, H. F., Mar............ 497

O'Connell, Matthew J., Oct...... 195
Oliver, William A., June........ 657
Osthues, Henry, June........... 657

Parke, Floyd B., Nov............ 246
Peck, Fayette H., July.......... 706
Phinney, Lorenzo N., June...... 657

Ransom, Harry B., Jan.......... 383
Reed, Charles S. R., Dec........ 313
Rice, William B., June.......... 657
Rood, Arthur, Dec.............. 313
Rowley, Ransom F., Sept........ 122
Russell, William P., Feb........ 430

Smith, Eugene A., Sept.......... 121
Smith, K. A., Sept.............. 120
Spencer, Herbert J., July........ 706

Taylor, Daniel L., Dec.......... 313
Turner, Jennie M., Nov.......... 246
Turner, John H., Nov........... 246

Van Alstyne, John L., Oct....... 195
Van Dyke, Charles M., Dec...... 313
Vebber, Frank M., Dec.......... 313
Voorhees, Sherman, June........ 656

Wales, Theron, A., Oct.......... 196
Webster, O. B., Oct............. 194
Willard, J. N., Dec.............. 313

### GENERAL INDEX.

ABDOMINAL CYST, etc., Robert T. Morris, April, page 503.
Air Hose, Deadly, May, page 577.
American College of Surgeons, Evening up with, Editorial, Feb., page 412.
Anaemia, Undescribed Cause of, etc., Pierce J. Candee, April, page 507.
Angina Pectoris, De Lancey Rochester, Jan., page 333.
Automobile, If you contemplate buying, Editorial, March, page 467.
Automobile Licensure, Editorial, June, page 631.
Automobile Statistics, May, page 575.

BATH HOUSES, First Free—in America, F. E. Fronczak, June, page 658.
Bone Grafting—Ununited Fracture of Tibia, Prescott Le Breton, Jan., page 344.

CANCER, Toxic Lymph Process, Frederick Gaertner, Sept., page 69.
Cancer, Editorial, July, page 687.
Cancer Campaign, Editorial, April, page 579.
Cancer Campaign, James H. Lewis, July, page 663.
Cancer of Colon, Diagnosis, Joseph Burke, Oct., page 137.
Cancer, Clinical Report of Cases Treated with Radium, John M. Lee, July, page 670.
Cancer, An Approach from the Nutritional Side, S. W. Little, July, page 679.
Carrier, Different Significance of Term, Editorial, March, page 463.
Commission Government, Editorial, Jan., page 359.

DECEPTION by Medical Assistance, Editorial, May, page 567.
Diabetic Gangrene, Mixed Infection Phylacogen in Treatment of, V. C. Pchellas, Aug., page 7.
Dispensary Abuse, Editorial, Nov., page 226.
Death Rates, Editorial, July, page 684.

ELECTROLYSIS in Dermatology, Jos. Spangenthal, Dec., page 278.
Empyema of Nasal Accessory Sinus, Joseph D. Lewis, May, page 562.
Epilepsy, New Serum Treatment, Wm. Held, June, page 618.
Ethics of Ante-Mortem Stimulation, Editorial, Dec., page 290.

FEEDING of Infants, Carl G. Leo-Wolf, Nov., page 201.
First Free Municipal Bath Houses in America, F. E. Fronczak, June, page 658.
Flies, Editorial, Sept., page 89.
Free Medicine, Reaction Toward, Editorial, Feb., page 408.

GANGRENE, Diabetic, Mixed Infection Phylacogen in Treatment of, V. C. Pchellas, Aug., page 7.
Goitre, etc., Robert T. Morris, April, page 503.
Goitre Problem, G. W. Cottis, March, page 456.

HARRISON LAW, After the—What?, Editorial, May, page 569.
Hip Amputation, etc., Robert T. Morris, April, page 503.
Human Corrosive Sublimate Tablet, Editorial, March, page 464.

IDIOT in Fiction, Editorial, March, page 464.
Industrial Independence, Editorial, Nov., page 223.
Infant Feeding, Carl G. Leo-Wolf, Nov., page 201.
Injuries of the Present War, W. W. Quinton, May, page 557.

JOURNALISTIC Laundry Work, Editorial, Sept., page 91.

LAW of Chance as Applied to Diagnostic Tests, Editorial, Aug., page 18.
Laws of N. Y., of Interest to Physicians, Sept. page 123, Nov. page 247.
Law, Harrison Anti-Narcotic, March, page 482; April, page 538; May, page 569.
Leukaemia, Chronic Lymphatic Complicated with Priapism, D. S. Hepburn, April, page 517.
Legislation, Professional Influence on, Editorial, March, page 465.

MALARIA, Historic Note, March, page 489.
Medical Economics, Live Issue in, Editorial, April, page 528.
Medical Economics, Practical Work in, Editorial, Oct., page 161.
Midwife, Editorial, April, page 522.
Military Service, Editorial, April, page 525.
Milk, Graded, May, page 575.
Milk Station, Aim of, F. E. Brundage, Dec., page 281.
Mineral Waters, Felix Von Oefele, Natural Nitrated, of New England, Sept., page 77.
Mineral Waters, Felix Von Oefele, Natural Silicated, of New England, Dec., page 286.
Mineral Waters, Felix Von Oefele, Thermal, Feb., page 405.
Mucous Polypi in the Nose, John C. Warbrick, Nov., page 221.
Municipal Bath Houses, F. E. Fronczak, June, page 658.

NASAL SEPTUM, Perforation of, J. C. Warbrick, May, page 563.
Natural Nitrated Mineral Waters of New England, Felix Von Oefele, Sept., page 77.
Natural Silicated Mineral Waters of New England, Felix Von Oefele, Dec., page 286.
New Field of Practice, T. D. Crothers, Nov., page 207.
New Serum Treatment for Epilepsy, Wm. Held, June, page 618.

OESOPHAGUS, Some Affections of, George F. Cott, Aug., page 10.
Old Saws and their Application to Medicine, Editorial, June, page 630.

PABULUM, W. Warren Britt, Nov., page 214.
Pancreatitis, Ross G. Loop, Sept., page 83.
Pellagra, Joseph Spangenthal, April, page 512.
Pensions for the Clergy, Why not for Physicians?, Editorial, June, page 626.
Perforation of Nasal Septum, J. C. Warbrick, May, page 563.
Phylacogen, Mixed Infection — in Treatment of Diabetes, V. C. Pchellas, Aug., page 7.
Polypi in the Nose, J. C. Warbrick, Nov., page 221.
Prejudices, Editorial, Sept., page 89.
Priapism—Chronic Lymphatic Leukaemia, D. S. Hepburn, Apr. page 517.
Psychic Muck-raking, Editorial, Dec., page 292.

QUARANTINE, Conditional vs. Unconditional, Albert J. Colton, Feb., page 399.

RADIUM, Clinical Report of Cancer Cases Treated With, John M. Lee, July, page 670.
Restrictive Legislation, Editorial, Oct., page 162.

Royalty, Decadent, Editorial, Jan., page 362.

Ruptured Spleen, E. W. Mulligan and Chas. W. Hennington, Sept., page 80

SEPTUM, Perforation of Nasal, John C. Warbrick, May, page 563.

Serum Treatment for Epilepsy, Wm. Held, June, page 618.

Skin Diseases, Urine in, H. C. Baum, Dec., page 267.

Spleen, Ruptured, E. W. Mulligan and Chas. W. Hennington, Sept., page 80

Status of Common, Weak and Cheap Drugs, Editorial, Aug., page 14.

Summer Medicine, Editorial, June, page 628.

TELEPHOTE, Editorial, Oct., page 166

Thermal Springs of Eastern Half of U. S., Felix Von Oefele, Feb., page 405.

Thymus Question, Chas. W. Hennington, June, page 611.

Thyroid Hyperplasia, R. P. Higgins, Dec., page 271.

Thyroid, see Goitre.

Tonsillar Disease, Inflammatory, Geo. F. Cott, Oct., page 148.

Transfusion of Blood, A. H. Noehren, Jan., page 347.

Tubercular Mortality, Editorial, Nov., page 227.

UNUNITED Fracture of Tibia—Bone Grafting, Prescott Le Breton, Jan., page 344.

Urine in Skin Diseases, H. C. Baum, Dec., page 267.

WAR, Editorial, Sept., page 92.

War, Injuries of Present, W. W. Quinton, May, page 557.

Woman Physician, Plea for, Editorial, Jan., page 357.

Word Blindness, Congenital, John C. Clemesha, Aug., page 1.

X-Ray Therapy, John Garratt, Feb., page 391.

**Reduction of Infections in Schools.** Rowland G. Freeman, Trans. Fourth Internat. Cong. on School Hyg., 1914, gives the following table:

| Disease | Incubation Period | Period during which exposed children should be kept out of school |
|---|---|---|
| Scarlet fever | Most often 3 to 4 days | 1st to 8th day |
| Measles | usually 11 days | 8th to 18th day |
| German measles | usually 17 days | 10th to 22d day |
| Chicken-pox | 14 to 16 days | 10th to 22d day |
| Whooping-cough | 7 to 14 days | 1st to 14th day |
| Mumps | Usually 17 to 20 days | 12th to 22d day |
| Diphtheria | 2 to 5 days | 1st to 5th day |

**Contradictory Findings in Wassermann Test.** A. L. Wolbarst, Interstate Med. Jour., Vol. 19, No. 2, 1915, has collected 85 cases in which three serologists agreed in 42%, differed more or less in 19% and differed grossly in 39%. Two serologists in 49 cases, agreed in 65%, differed in 23% and contradicted each other in 12%. He advocates, therefore, a uniform method, preparation and distribution of reagents from a central station and careful selection of examiners, although it is implied that the examiners were supposedly competent. (Note: We would suggest a further conclusion, namely that under existing conditions, the Wassermann reaction is, for practical purposes, at best, no more diagnostic than clinical signs as observed by competent men).

